# Newborn with Gastroschisis associated with Limb Anomalies

**DOI:** 10.21699/jns.v6i2.595

**Published:** 2017-04-15

**Authors:** Mark Lubinsky

**Affiliations:** Not affiliated after retirement

**Dear Sir**

The patients reported with gastroschisis and limb anomalies [1] appear to represent a known association with amyoplasia, a form of congenital joint contractures. This combination is consistent with vascular effects on the developing spinal cord with amyoplasia [2] and a vascular pathogenesis for gastroschisis, where considerable evidence should be convincing [3]. Although, as the author notes, the limb deformities do not need immediate attention, there is some urgency, since “there appears to be a 4-month window after birth where physical therapy is particularly useful” [2].

## Footnotes

**Source of Support:** Nil

**Conflict of Interest:** None
